# Comparative Characterization of Leukocyte-Rich Platelet-Rich Plasma (L-PRP) and Injectable Platelet-Rich Fibrin (i-PRF): A Laboratory Study

**DOI:** 10.3390/cells15100886

**Published:** 2026-05-13

**Authors:** André Vinicius Saueressig Kruel, Mariângela Ferreira, Daiane Agostini, Cristiano Valter Diesel, Marcelo Queiroz, Carlos Roberto Galia, Guilherme Liberato da Silva, Stephany Huber, Fernanda Majolo

**Affiliations:** 1 Clínica PROREGEN, Bento Gonçalves 95700-066, RS, Brazil; maricferreira@gmail.com (M.F.); daianeagostini.med@gmail.com (D.A.); 2 Programa de Pós-Graduação em Biotecnologia (PPGBiotec), Universidade do Vale do Taquari (Univates), Lajeado 95914-014, RS, Brazil; fmajolo@univates.br; 3 Programa de Pós-Graduação em Ciências Médicas (PPGCM), Universidade do Vale do Taquari (Univates), Lajeado 95914-014, RS, Brazil; gibaliberato@univates.br; 4 Hospital Moinhos de Vento, Porto Alegre 90560-032, RS, Brazil; cristianodiesel@gmail.com; 5 Hospital Israelita Albert Einstein, São Paulo 05651-901, SP, Brazil; marcelo.queiroz@einstein.br; 6 Hospital de Clínicas de Porto Alegre, Porto Alegre 90410-000, RS, Brazil; carlosgalia.clinica@gmail.com; 7 Orthoregen Medicina, Ensino e Pesquisa, Indaiatuba 13334-170, SP, Brazil; ioc.orthoregen@gmail.com

**Keywords:** platelet-rich plasma, platelet-rich fibrin, growth factors, regenerative medicine, blood platelets

## Abstract

**Highlights:**

**What are the main findings?**

- Leukocyte-rich PRP delivers a markedly higher platelet concentration and greater immediate total PDGF-BB content, whereas i-PRF provides a larger volume and a higher absolute leukocyte payload.
- Donor sex, abdominal circumference, and comorbidity status influence product volume and total leukocyte yield, but the preparation protocol remains the primary determinant of platelet concentration.

**What are the implications of the main findings?**

- PRP and i-PRF exhibit distinct laboratory profiles and may not be interchangeable; product selection should be guided by the desired cellular and growth factor delivery profile.
- Well-designed randomized clinical trials are required to determine whether these in vitro differences translate into tissue-specific clinical superiority.

**Abstract:**

Introduction: Orthobiologics such as Platelet-Rich Plasma (PRP) and Injectable Platelet-Rich Fibrin (i-PRF) have emerged as promising tools in regenerative medicine. However, the lack of methodological standardization and the still limited comparative characterization between these products represent significant barriers to their optimized clinical application. This comparative laboratory study aimed to characterize and differentiate PRP and i-PRF, focusing on their cellular composition, obtained volume, and total Platelet-Derived Growth Factor (PDGF-BB) content. Materials and Methods: This study was conducted with 34 individuals meeting standard blood donation criteria. Peripheral blood samples were collected from all participants. PRP was obtained using a modified double-spin centrifugation protocol, whereas i-PRF was prepared using a modified low-speed centrifugation technique. Cellularity (platelet and leukocyte counts), final produced volume, and total PDGF-BB content were assessed using complete blood count analysis and an enzyme-linked immunosorbent assay (ELISA), respectively. Statistical analysis was performed using Linear Mixed Models (LMMs). Results: Both protocols resulted in significant increases in platelet and leukocyte concentrations compared to baseline values. PRP showed significantly higher platelet and leukocyte concentrations compared with i-PRF, as well as markedly higher PDGF-BB levels. In contrast, i-PRF yielded a substantially greater final volume and enabled a higher absolute delivery of total leukocytes, whereas PRP delivered a greater absolute number of platelets. In exploratory analyses, female sex, the presence of comorbidities, and increased abdominal circumference were associated with variations in product volume and cellular composition. Discussion: These findings indicate that PRP and i-PRF exhibit distinct biological profiles in terms of cellularity, volume, and total PDGF-BB content. Whether these laboratory differences translate into distinct clinical outcomes remains unknown. The results should therefore be viewed as hypothesis-generating: they suggest that PRP and i-PRF may not be interchangeable, and that future randomized clinical trials are needed to define product-specific indications based on the target tissue and desired biological mechanism.

## 1. Introduction

Platelet-Rich Plasma (PRP) is a traditional orthobiologic [1,2,3] with more than three decades of clinical use. It is defined as the plasma fraction (containing anticoagulant) with a platelet concentration higher than physiological levels [4,5]. Its clinical applicability is supported by high-level scientific evidence across several musculoskeletal conditions, particularly knee osteoarthritis [6,7] and tendinopathies [8,9], as well as other indications [6].

Given that patient-specific factors (e.g., sex, metabolic status) are known to influence baseline hematology, an exploratory analysis of these variables was included to assess their potential impact on final product composition, thereby informing future patient selection or protocol customization. However, technical variability associated with the lack of methodological standardization represents one of the main factors contributing to heterogeneity in PRP composition, and ultimately leading to poor reproducibility of findings, hindering comparative studies and meta-analyses, and results in inconsistent clinical outcomes, providing arguments for critics of PRP therapies [10]. In this context, our research group has previously developed solutions such as classification proposals for PRP preparation techniques [11].

The growing interest in orthobiologics and their therapeutic potential has driven the development of a second generation of PRP-derived products, including Injectable Platelet-Rich Fibrin (i-PRF) [12]. These preparations offer immediate advantages, such as reduced costs due to simplified centrifugation systems and the elimination of anticoagulants [13], which may theoretically promote a more physiological tissue repair process [14].

Nevertheless, differences in the characterization and protocolization of these products, as well as distinctions in the resulting preparations, are not clearly delineated in the scientific literature. This gap represents a barrier to the proper development of future clinical trials aimed at elucidating the specific benefits and clinical indications of each orthobiologic.

Regarding the characterization of these products, the Platelet-Derived Growth Factor (PDGF) is selected frequently as a marker in platelet concentrate evaluations [15]. It is an important cytokine and marker of platelet function stored in the alpha granules of this critical cellular fragment involved in the initiation of tissue repair processes. PDGF comprises a family of disulfide-linked dimeric proteins that play a fundamental role in regenerative medicine. This family includes four homodimeric isoforms (PDGF-AA, PDGF-BB, PDGF-CC, and PDGF-DD) and one heterodimeric isoform (PDGF-AB). Among these, PDGF-BB stands out as the most versatile and potent subtype, capable of activating both PDGF receptors and inducing more robust biological responses. We therefore present a comparative laboratory study between two distinct and established protocols, leukocyte-rich PRP (L-PRP) and i-PRF, both obtained from the same participating donor. Hereafter, whenever the term PRP is used, it refers to a leukocyte-rich formulation within the context of the present protocol. The study focuses on differences in cellularity, volume, and quantitative analysis of Platelet-Derived Growth Factor (PDGF-BB).

## 2. Materials and Methods

The study was conducted at the Proregen Clinic—Regenerative Orthopedics, located in Bento Gonçalves, RS, Brazil.

### 2.1. Ethical Aspects

This project was registered on the AGHUse Pesquisa platform and Plataforma Brasil and approved by the Research Ethics Committee of the Hospital de Clínicas de Porto Alegre under approval number 6,640,941 (CAAE: 74707623.8.0000.5327); Approval date: 16 May 2024). The researchers conducted the study in accordance with ethical principles, ensuring data confidentiality and participant privacy, in compliance with Brazilian National Health Council Resolutions CNS 466/2012 and CNS 510/2016, as well as all applicable regulations and legislation. In addition, the researchers adhered to the requirements of Brazilian Law No. 13,709, dated 14 August 2018, regarding the processing of sensitive personal data used in the research. Informed consent was obtained from all subjects involved in the study.

### 2.2. Inclusion and Exclusion Criteria

The sample included adult individuals of both sexes, aged between 18 and 70 years, who met all internationally recognized basic blood donation criteria [16]. These criteria included a minimum body weight of 50 kg, adequate rest (at least 6 h of sleep in the previous 24 h), having eaten prior to donation, and being in good general health. Individuals who did not meet blood donation criteria or who presented abnormal erythrogram values (hemoglobin < 13.0 g/dL for men and <12.0 g/dL for women; hematocrit < 39% for men and <36% for women, in accordance with WHO guidelines) after blood collection were excluded from the study.

### 2.3. Variables of Interest

In addition to general demographic information, the collected variables included anthropometric data (body weight, height, body mass index [BMI], and abdominal circumference), complete blood count, and habitual medication use. Furthermore, product-related parameters such as cellularity, produced volume, and PDGF quantification are detailed below.

### 2.4. Peripheral Blood Collection

From each participant, 66 mL of whole blood was collected via peripheral venipuncture and distributed into nine BD Vacutainer^®^ (Becton, Dickinson and Company, East Rutherford, NJ, USA) tubes:

- Four tubes containing ACD (8.5 mL capacity, containing 1.5 mL of acid citrate dextrose): 7 mL of blood per tube, totaling 28 mL for PRP production;
- Four dry tubes without anticoagulant or clot activator (9 mL capacity): 9 mL of blood per tube, totaling 36 mL for i-PRF production;
- One EDTA tube (2 mL): used for baseline complete blood count, allowing for comparison of platelet and leukocyte levels with those obtained in PRP and i-PRF.

The selection of tubes with similar capacities but different blood volumes was based on their availability for Cell Processing Centers in Brazil and compliance with Brazilian Health Regulatory Agency (ANVISA) standards for open systems used in PRP and i-PRF preparation.

### 2.5. Preparation of Leukocyte-Rich Platelet-Rich Plasma

The PRP preparation protocol followed the modified technique described by Amable et al. [17] and validated by Huber et al. [18]. After peripheral blood collection, the blood-containing tubes were centrifuged at 300× *g* for 5 min to separate red blood cells, the leukocyte layer (buffy coat), and plasma. After the first centrifugation, plasma was collected together with the buffy coat using a 14 G needle, minimizing red blood cell contamination. Hereafter, whenever the term PRP is used, it refers to a leukocyte-rich formulation within the context of the present protocol.

This content (plasma + buffy coat + platelets) was transferred to a sterile 15 mL Falcon tube and subjected to a second centrifugation at 700× *g* for 17 min, resulting in platelet and leukocyte concentration in a pellet at the bottom of the tube. From the total volume, 80% of the upper plasma layer, corresponding to platelet-poor plasma, was removed, and the remaining 20% (cell pellet—PRP) was homogenized and sent for cell counting. Aliquots of 500 µL were stored at −80 °C for PDGF quantification. No sample was stored for longer than 180 days. All processing steps were performed in a class IIA biological safety cabinet, in accordance with Brazilian Health Regulatory Agency (ANVISA) Resolution RDC 508/2021.

### 2.6. Preparation of Injectable Platelet-Rich Fibrin

i-PRF was produced using a modified low-speed centrifugation technique [19]. Briefly, the four tubes containing whole blood without anticoagulant were immediately centrifuged at 60× *g* for 5 min to separate serum (still in liquid form), red blood cells, and buffy coat.

The tube cap was disinfected with 70% ethyl alcohol and punctured using a 14 G catheter. The first 2 mL were discarded, and the milliliter closest to the red blood cell layer (buffy coat) was aspirated using the needle. A 100 µL aliquot of this fraction was transferred to an EDTA tube for platelet and leukocyte quantification, and three 500 µL aliquots were stored at −80 °C for subsequent growth factor analysis.

To ensure reproducibility and minimize operator-dependent variability, a standardized ‘Fast-Handling’ protocol was employed. The time elapsed from the initiation of blood collection to the start of centrifugation was recorded and kept below 180 s. For i-PRF collection, the needle tip was held 1 mm from the red blood cell layer, and the buffy-coat fraction was collected. All procedures were performed by the two trained technicians to ensure interface consistency and avoid cross-contamination of layers.

Because i-PRF undergoes spontaneous fibrin polymerization in the absence of anticoagulant, the cellular counts reported herein represent the liquid/suspended fraction obtained immediately after centrifugation. Cells entrapped within the fibrin matrix are not captured by this method; therefore, the measured platelet and leukocyte concentrations likely underestimate the total cellular payload of the i-PRF preparation.

### 2.7. Evaluation of Both Products

**Figure 1:**
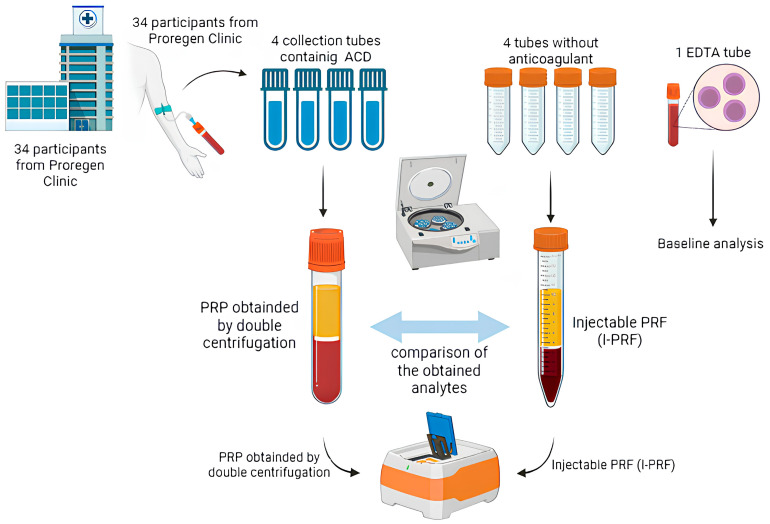
Summary of blood collection procedures to produce blood-derived orthobiologics.

The parameters used for PRP and i-PRF validation were as follows: (i) platelet count; (ii) leukocyte count; (iii) produced volume; and (iv) concentration of platelet-derived growth factor (PDGF-BB), assessed by using an enzyme-linked immunosorbent assay (ELISA). Platelet and leukocyte recovery was evaluated using complete blood count analysis, in which baseline values for each individual were compared with those obtained in PRP and i-PRF. Produced volume and overall cellular recovery (platelet and leukocyte) were also assessed, which is particularly relevant for clinical correlation regarding absolute cell delivery in therapeutic protocols (Figure 1).

### 2.8. Enzyme-Linked Immunosorbent Assay (ELISA)

Accurate quantification of PDGF-BB, particularly through an enzyme-linked immunosorbent assay (ELISA), is essential for assessing the quality and efficacy of platelet-based preparations used in regenerative procedures. For PDGF-BB quantification in PRP and i-PRF, samples were thawed in a water bath at 37 °C. PDGF-BB levels were quantified using a commercially available ELISA kit (R&D Systems, Inc. (Bio-Techne), Minneapolis, MN, USA) with reactivity for human samples (Fine Test, Interprise), which is available on the national market. All analyses were performed at the Laboratory Research Unit of the Experimental Research Center of the Hospital de Clínicas de Porto Alegre (HCPA).

Technical issues related to i-PRF fibrin clot formation precluded reliable multiplex cytokine profiling; the corresponding analytical limitations are addressed in the Discussion.

Briefly, the assay is based on sandwich ELISA technology. An anti–PDGF-BB antibody was pre-coated onto a 96-well microplate. A biotin-conjugated anti–PDGF-BB antibody was used as the detection antibody. Standards and test samples (100 µL) were added to the wells and incubated for 90 min at room temperature.

After incubation, unbound conjugates were removed by washing with wash buffer. Subsequently, 100 µL of the biotinylated detection antibody was added to bind the target protein captured by the coated antibody, followed by incubation for 1 h. The plate was then washed three times.

After the removal of unbound conjugates, 100 µL of horseradish peroxidase (HRP)–streptavidin was added and incubated for 30 min, followed by five washing cycles. Subsequently, 90 µL of tetramethylbenzidine (TMB) substrate was added to visualize the enzymatic reaction and incubated for 15 min.

Finally, 50 µL of stop solution (sulfuric acid) was added to each well. The enzymatic reaction produced a blue color that turned yellow upon addition of the stop solution. Absorbance was measured at 450 nm using a microplate reader. The concentration of the target protein in each sample was calculated by constructing a standard curve, with analyte concentration being proportional to the measured absorbance values.

### 2.9. Data Analysis

Collected data were stored in a Microsoft Excel spreadsheet. Statistical analyses were performed using JAMOVI software version 2.3 (Accessed date: 25 July 2025) and the R Core Team environment version 4.1 (Accessed date: 25 July 2025). Data normality was assessed using the Kolmogorov–Smirnov and Shapiro–Wilk tests. Continuous variables analyzed in independent groups were expressed as the mean ± standard deviation. Categorical variables were presented as number and percentage [n (%)], and nominal categorical variables were analyzed using the Chi-square (χ^2^) test with continuity correction or Fisher’s exact test, as appropriate.

Because multiple samples (baseline whole blood, PRP, and i-PRF) were obtained from each participant, the data exhibit a repeated-measures, within-donor dependence structure. To account for this, all primary comparative analyses were performed using Linear Mixed Models (LMMs) with a random intercept per participant (ID). This approach explicitly models the correlation among repeated observations from the same individual, thereby yielding unbiased standard errors and valid hypothesis tests.

The choice of distribution family and link function was guided by the nature of each outcome variable and formally compared using the Akaike Information Criterion (AIC). After testing Gamma (log and identity links), Negative Binomial, and Gaussian (identity) distributions, the linear mixed model (LMM) with Gaussian distribution and identity link consistently provided the lowest AIC, satisfactory residual diagnostics, and adequate convergence for the following outcomes: total PDGF-BB content, platelet count, leukocyte count, volume, total platelet yield, and total leukocyte yield. For these outcomes, results are reported as mean differences (β coefficients) with 95% confidence intervals and *p*-values derived from the t-distribution.

The full model included the following explanatory variables: Group (Baseline/PRP/i-PRF), Sex (Female/Male), Comorbidity (No/Yes), Alcohol consumption (No/Yes), Abdominal circumference (cm), and Age (years). For each outcome, the full model was fitted first; the best-fitting model was then selected by backward elimination based on AIC minimization, with the primary predictor Group retained in all models regardless of significance. Post-hoc comparisons. When the overall effect of Group was significant (χ^2^ test), pairwise comparisons between the three levels (Baseline, PRP, i-PRF) were performed using estimated marginal means. To control the family-wise error rate, Bonferroni correction was applied to the three pairwise contrasts within each outcome.

Model adequacy was assessed via simulation-based residual diagnostics, including Q–Q plots, residual vs. predicted plots, and tests for overdispersion, zero-inflation, and heteroscedasticity. Multicollinearity among fixed effects was evaluated using the variance inflation factor (VIF); values exceeding 5 were considered indicative of problematic collinearity (this threshold replaces the overly conservative criterion used in the initial submission). Model convergence was verified by confirming that gradient calculations remained below default tolerance thresholds. Full model summaries (Fixed-effect coefficients, standard errors, 95% confidence intervals, and *p*-values) are reported in results tables.

Given the exploratory, laboratory-based nature of the study and its hypothesis-generating intent, formal correction for multiplicity across the seven primary outcomes was not applied to the main analyses, to avoid excessive Type II error inflation. All *p*-values are reported without cross-outcome adjustment; a *p*-value < 0.05 was considered nominally significant. As a sensitivity check, we verified that the key comparative findings (Group effects for PDGF-BB, platelet concentration, and total leukocyte yield) remain significant under a Bonferroni-adjusted threshold of *p* < 0.007 (corresponding to seven primary outcomes).

To account for the inherent difference in initial blood volumes between the PRP protocol (28 mL) and the i-PRF protocol (36 mL), two complementary metrics were employed. First, absolute total platelet and leukocyte yields were calculated by multiplying the final product volume by the respective cell concentration, representing the complete cellular payload delivered by each preparation. Second, percentage volume recovery was defined as (Final Volume (mL)/Initial Blood Volume (mL) × 100, enabling a normalized comparison of processing efficiency independent of the starting volume. These metrics are reported alongside concentration-based measures. Because the recovery values are paired within donors and their distribution could not be assumed normal, the comparison between PRP and i-PRF recovery was performed using the Wilcoxon signed-rank test for dependent samples. A two-sided *p*-value < 0.05 was considered significant for all analyses.

The sample size calculation was performed using the GPower 3.1 software post hoc with the “Means: Difference between two dependent means (matched pairs)” test. An effect size ω of 0.50 (50%), a test power of 80%, and an alpha level of 0.05 were used to reduce type I and type II errors of the primary outcome PDGF-BB, yielding a total sample of 34 individuals for data collection. The achieved power was 80.7%, which supports the validity of our comparative findings.

## 3. Results

A total of 36 participants were initially selected for the study; however, two were excluded: one due to a diagnosis of diarrheal syndrome with onset on the day of blood collection, and another due to hypoferritinemia. Thus, 34 individuals who fulfilled all donation criteria were included in the analysis, of whom 26 (76.5%) were female and 8 (23.5%) were male. In addition, 33 participants (97.1%) were Caucasian, and only one self-identified as Black (2.9%).

Regarding BMI categories, normal-weight and overweight individuals were the most frequent, whereas obesity was less prevalent. Only one participant reported being a smoker. Notably, half of the participants reported alcohol consumption, and 70.6% reported regular use of medications, while 35.3% presented at least one comorbidity (Table 1).

Among the comorbidities observed in the total sample, systemic arterial hypertension was the most prevalent, affecting six individuals (17.6%), followed by hypothyroidism and migraine, each with two cases (5.88%). Asthma, dyslipidemia, fibromyalgia, Nutcracker syndrome, and pelvic varices were each reported in one individual (2.94%). The most frequently used medications were antidepressants (venlafaxine, escitalopram, duloxetine, and fluoxetine), used by 29.4% of the study population; antihypertensive agents (losartan, hydrochlorothiazide, olmesartan, enalapril, and propranolol), used by 26%; and contraceptives or estradiol, used by 20% of participants (Table 1).

No significant association was observed between sex and BMI category (χ^2^ = 6.08; df = 4; *p* = 0.16), nor between sex and the presence of comorbidities (χ^2^ = 0.32; df = 1; *p* = 0.41). The continuous variables measured in eligible participants generally demonstrated higher mean platelet and leukocyte counts in PRP, followed by i-PRF, and subsequently by baseline values (Table 2). The i-PRF showed a percentage volume recovery approximately twice that of PRP (Z = 5.08; *p* < 0.001).

### 3.1. Baseline, Platelet-Rich Plasma (PRP), and Injectable Platelet-Rich Fibrin (i-PRF)

The assumptions of all regression models were verified. Diagnostic plots indicated no severe violations of linearity, homoscedasticity, or normality of residuals. Multicollinearity was not a concern in the final models, as all variance inflation factors (VIFs) were below 2.0.

**Figure 2:**
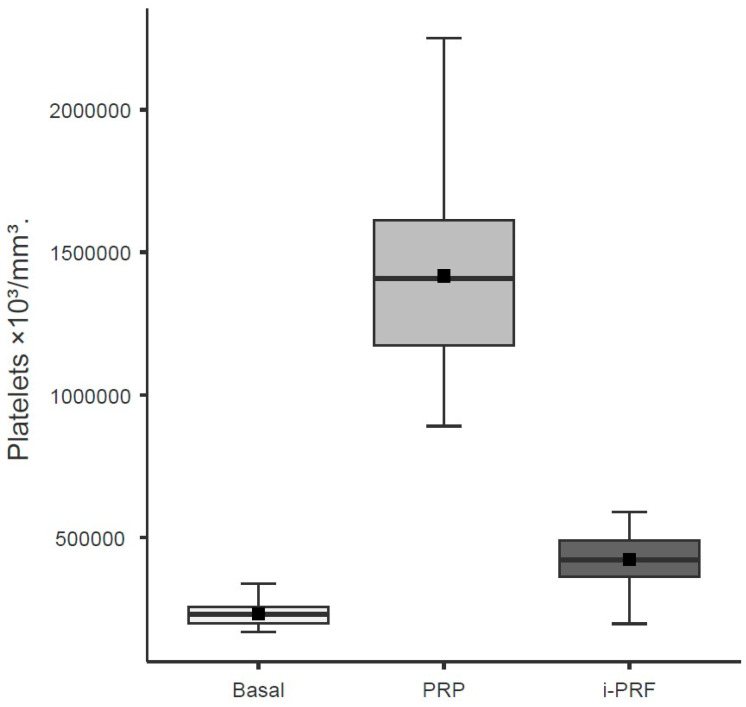
Platelet concentrations in baseline whole blood, leukocyte-rich PRP, and i-PRF. Boxes represent the interquartile range (IQR), horizontal lines inside boxes indicate the median, black squares indicate the mean, and whiskers extend to the most extreme data points within 1.5× IQR. Units: platelets ×10^3^/mm^3^. All pairwise comparisons between groups were significant (*p* < 0.001; LMM regression).

Based on LMM analysis, the variable Group (χ^2^ = 538.43; df = 2; *p* < 0.001) showed a significant effect on platelet count. Regarding platelet levels across groups, PRP presented the highest values, followed by i-PRF, and finally the baseline sample (Table 3; Figure 2).

**Figure 3:**
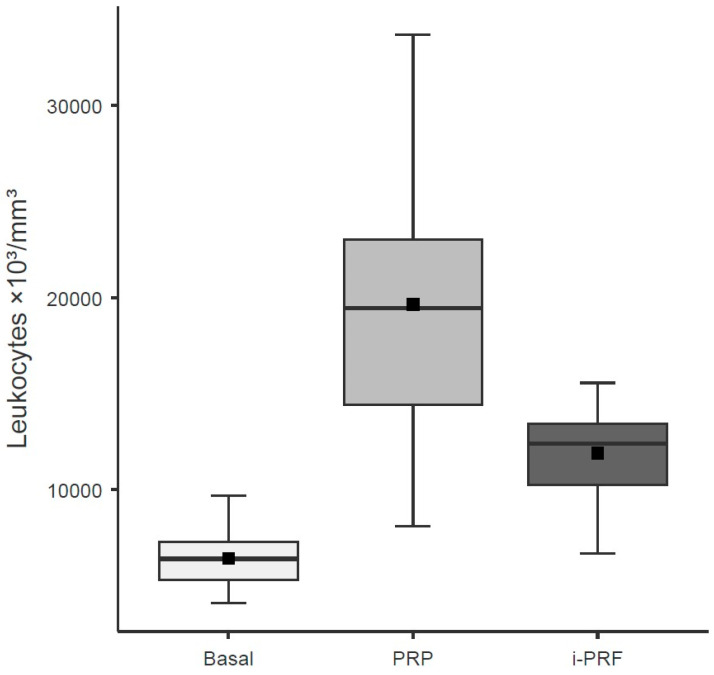
Leukocyte concentrations in baseline whole blood, leukocyte-rich PRP, and i-PRF. Boxes represent the interquartile range (IQR), horizontal lines inside boxes indicate the median, black squares indicate the mean, and whiskers extend to the most extreme data points within 1.5 × IQR. Units: leukocytes ×10^3^/mm^3^. All pairwise comparisons between groups were significant (*p* < 0.001; LMM regression).

Regarding leukocyte count, only the variable Group, which represents the type of blood-derived sample analyzed (baseline whole blood, PRP, i-PRF), showed a significant effect (χ^2^ = 311.30; df = 2; *p* < 0.001). PRP exhibited the highest leukocyte counts, with a mean difference of 13,216.5 leukocytes compared with baseline and a mean difference of 5472 leukocytes compared with i-PRF (Table 4; Figure 3).

### 3.2. Platelet-Derived Growth Factor–BB Isoform (PDGF-BB) and Volume (PRP vs. i-PRF)

**Figure 4:**
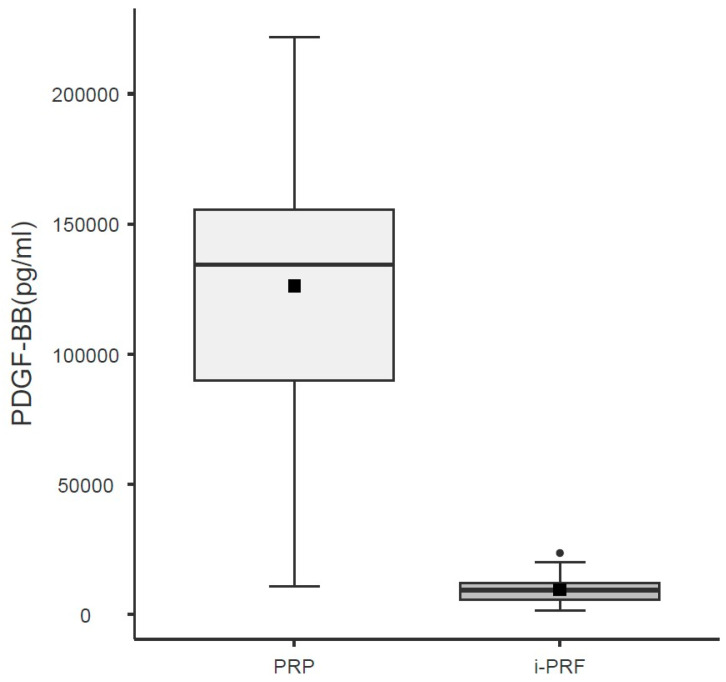
Total PDGF-BB content in leukocyte-rich PRP and i-PRF. Boxes represent the interquartile range (IQR), horizontal lines inside boxes indicate the median, black squares indicate the mean, and whiskers extend to the most extreme data points within 1.5 × IQR. Units: PDGF-BB pg/mL. The difference between groups was significant (*p* < 0.001; LMM regression).

Regarding PDGF-BB levels, the regression analysis demonstrated that the variable Group (χ^2^ = 199.38; df = 1; *p* < 0.001) had a significant effect. The i-PRF group was associated with a significant reduction in the expected level of the outcome variable (PDGF-BB) when compared with the reference group (PRP) (Table 5; Figure 4).

**Figure 5:**
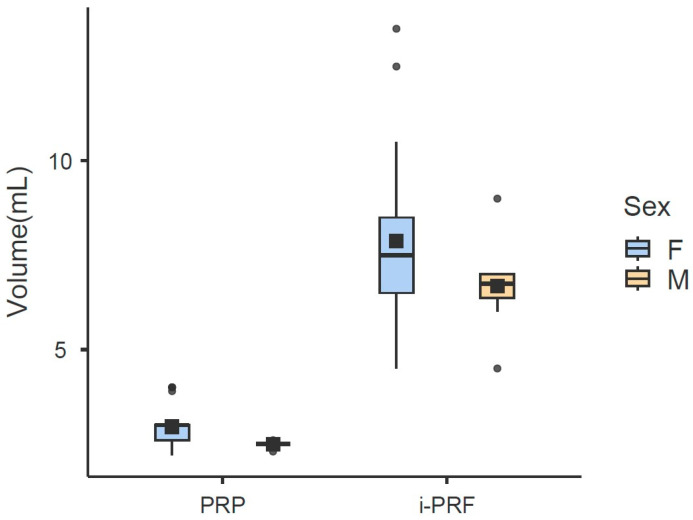
Final volume (mL) of leukocyte-rich PRP and i-PRF, stratified by sex (F = Female; M = Male). Boxes represent the interquartile range (IQR), horizontal lines inside boxes indicate the median, black squares indicate the mean, and whiskers extend to the most extreme data points within 1.5 × IQR. Units: mL. Both product type (PRP vs. i-PRF) and sex were significant predictors of volume (*p* < 0.001 and *p* = 0.009, respectively; LMM).

Regarding volume (mL), the variables Group (χ^2^ = 286.06; df = 1; *p* < 0.001), Sex (χ^2^ = 7.8; df = 1; *p* = 0.009), Abdominal Circumference (χ^2^ = 8.93; df = 1; *p* = 0.006), and Comorbidity (χ^2^ = 5.99; df = 1; *p* = 0.02) were found to have a significant effect. The estimator for the Sex variable was negative (−1.32), indicating that men present, on average, a volume 1.32 mL lower compared to women (reference group). The i-PRF group showed an average of 4.75 mL more than the PRP group. This volume difference reflects both the distinct initial collection (36 mL for i-PRF versus 28 mL for PRP) and different concentration rates: PRP concentrated 10.14% of total blood (average of 2.84 mL from 28 mL), while i-PRF concentrated 20.11% (average of 7.6 mL from 36 mL). The estimator for the Abdominal Circumference variable was significantly positive (0.04), indicating that for every 1 cm increase in Abdominal Circumference, there is an average increase of 0.04 mL in Volume (Table 6; Figure 5). In exploratory analyses, individuals with at least one comorbidity presented, on average, a 0.93 mL higher volume compared to those without this condition. However, given the small size and heterogeneity of the comorbidity group, this finding should be interpreted with caution.

### 3.3. Total Platelets and Total Leukocytes (Platelet-Rich Plasma [PRP] vs. Injectable Platelet-Rich Fibrin [i-PRF])

**Figure 6:**
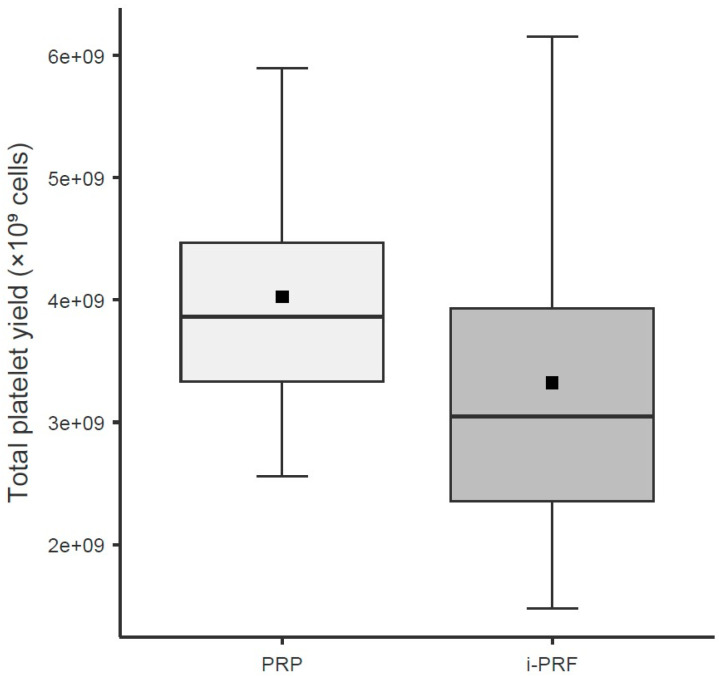
Total platelet yield (cells) in leukocyte-rich PRP and i-PRF. Boxes represent the interquartile range (IQR), horizontal lines inside boxes indicate the median, black squares indicate the mean, and whiskers extend to the most extreme data points within 1.5 × IQR. Units: Total platelet yield (×10^9^ cells); PRP delivered significantly higher total platelets than i-PRF (*p* < 0.001; LMM regression).

Regarding total platelet counts, the variable Group showed a significant effect (χ^2^ = 23.67; df = 1; *p* < 0.001) (Table 7; Figure 6).

Regarding total leukocyte counts, it was possible to verify that the variables Group (χ^2^ = 92.58; df = 1; *p* < 0.001), Comorbidity (χ^2^ = 6.75; df = 1; *p* = 0.01), and Abdominal Circumference (χ^2^ = 10.6; df = 1; *p* = 0.003) showed significant effects.

The presence of comorbidity was associated with an increase in total leukocyte counts compared to the group without comorbidities. However, the comorbidity group comprised various conditions, and the small sample size precludes definitive conclusions about biological causality. In addition, the i-PRF group is associated with a significant increase in the expected level of total leukocytes compared to the reference group (PRP).

**Figure 7:**
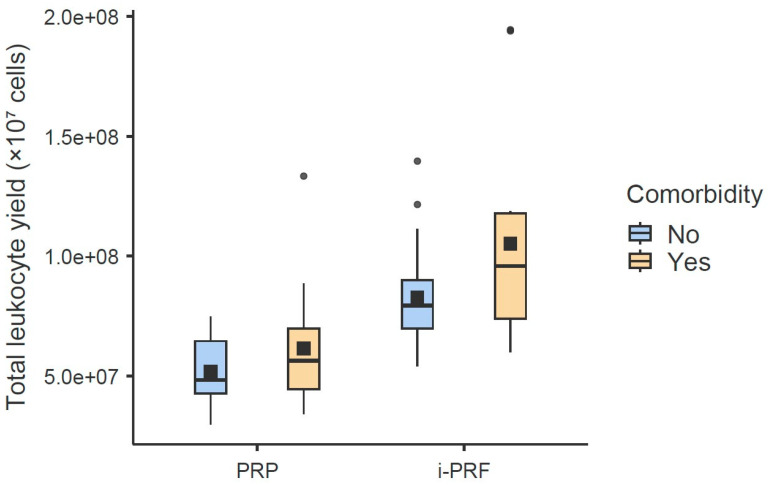
Total leukocyte yield (cells) in leukocyte-rich PRP and i-PRF, stratified by presence of comorbidity (No/Yes). Boxes represent the interquartile range (IQR), horizontal lines inside boxes indicate the median, black squares indicate the mean, and whiskers extend to the most extreme data points within 1.5 × IQR. Units: Total leukocyte yield (×10^7^ cells). i-PRF delivered significantly higher total leukocytes than PRP (*p* < 0.001); comorbidity was also associated with higher total leukocyte yield (*p* = 0.01; LMM).

A similar significant increase was also observed for abdominal circumference, suggesting an increase in the total leukocyte rate for each 1 cm increase in abdominal circumference (Table 8; Figure 7).

## 4. Discussion

Following the analysis of samples from 34 participants and the characterization of the experimental protocols, this laboratory study demonstrated that both platelet-rich plasma (PRP) and injectable platelet-rich fibrin (I-PRF) significantly increased platelet concentration when compared with baseline values. However, PRP consistently yielded a higher platelet concentration than i-PRF, corroborating previous reports describing PRP as a more efficient platelet-concentrating system [18,19]. These findings provide a useful reference for future protocol standardization, whether using open systems in cell processing centers or commercially available closed kits.

None of the independent variables evaluated sex, comorbidities, alcohol consumption, abdominal circumference, or age, significantly influenced platelet concentration, reinforcing that the preparation protocol itself is the primary determinant of platelet yield. This highlights the importance of protocol selection rather than patient-related factors when aiming to optimize platelet concentration in blood-derived orthobiologics.

Leukocyte concentration also increased following blood processing, with PRP and i-PRF both presenting higher leukocyte counts than baseline. The PRP protocol employed prioritized buffy coat harvesting, which explains its higher leukocyte concentration. Specifically in tendinopathy, where the rate of cytokine release and the presence or absence of leukocytes may influence tissue repair, this comparative analysis between PRP and i-PRF may have clinically relevant implications [9,13,14]. In tendinopathies, leukocyte-rich preparations—especially those containing monocytes—have been associated with improved healing responses [8,9]. Although PRP demonstrated higher leukocyte concentration than i-PRF, the clinical superiority of either product remains to be determined and should be addressed in future standardized clinical trials.

Consistent with the higher platelet concentration observed in PRP, PDGF-BB levels measured by ELISA were markedly higher in PRP than in i-PRF, in agreement with previous investigations [20]. Importantly, this difference cannot be explained solely by platelet concentration, suggesting that the distinct biological behavior of these products plays a critical role. PRP is characterized by rapid platelet degranulation and immediate total PDGF-BB release, facilitating early detection of PDGF-BB. In contrast, i-PRF forms a fibrin matrix that promotes slower, more physiological cytokine release, which may limit early PDGF-BB detection by ELISA but potentially prolong biological activity in vivo [14]. While the measured concentration of PDGF-BB was significantly higher in the PRP group (*p* < 0.05), these values should be interpreted as timepoint-specific measurements. Given that i-PRF forms a polymerized fibrin secondary to the absence of anticoagulants, a significant portion of the growth factor load may remain sequestered within the matrix at the time of assay. Therefore, these results characterize the immediate release potential rather than the total cumulative biological availability over the lifespan of the clot. Future basic science studies may help better understanding of this potential mechanism.

From a practical perspective, blood-derived orthobiologics may require different volumes depending on the target tissue. While PRP typically produces smaller volumes, i-PRF offers a higher yield with simpler and faster processing, representing a practical clinical advantage. Sex-related differences in volume production were observed, likely reflecting physiological differences in hematocrit, as previously described. Abdominal circumference and comorbidity status were also associated with volume variations, possibly due to differences in plasma volume and hematological profiles.

The predominantly female and Caucasian composition of our cohort, although reflecting the demographic profile that frequently seeks orthopedic treatments in our region, raises important questions regarding the generalizability of our findings. Sex emerged as a significant predictor of product volume, with male individuals yielding, on average, 0.83 mL less than their female counterparts. This difference may be attributed to well-documented physiological hematological variations between sexes, including differences in hematocrit, plasma volume, and baseline platelet counts [21,22].

Although platelet and leukocyte concentrations were not significantly influenced by sex in our analysis, it is plausible that differences in platelet activation or leukocyte subset distribution parameters not assessed in this study, could modify the final biological profile of the products. For instance, some studies suggest that platelets from female individuals may exhibit different degranulation patterns in response to certain stimuli. Furthermore, leukocyte composition (neutrophil-to-lymphocyte ratio) may vary between sexes and potentially influence the inflammatory microenvironment following application.

These considerations are particularly relevant to regenerative medicine, where sex-specific biological responses have been observed in other therapeutic modalities. Therefore, our volume-related findings should be interpreted with caution in populations with different sex distributions, and future studies should include sex-balanced samples to validate our results and explore potential functional differences in the concentrated cells.

When absolute cellular delivery is considered, PRP provided a higher total number of platelets, whereas i-PRF delivered a greater total number of leukocytes. These findings suggest that PRP delivers a higher platelet payload in vitro, which may be relevant in scenarios requiring rapid total PDGF-BB availability, whereas i-PRF provides a sustained-release scaffold. Given the growing evidence supporting the role of leukocytes—particularly within a fibrin matrix—in modulating inflammation and tissue regeneration, i-PRF warrants further investigation in clinical scenarios such as tendinopathies [8,9].

It is important to distinguish between the biological potential of the platelet concentrates and the outcomes dictated by their specific preparation protocols. In this study, the variations in starting blood volume and the presence of anticoagulants in the PRP group represent inherent procedural differences. While these factors introduce analytical confounding, they reflect the real-world clinical application of these products. Readers should interpret the observed contrasts as performance benchmarks of the protocols rather than immutable biological distinctions between the two mediums.

Additionally, the presence of comorbidities was associated with increased total leukocyte delivery, reflecting expected immune responses in eligible participants with chronic conditions. While this observation aligns with the concept of low-grade inflammation in chronic conditions, our comorbidity group was small and heterogeneous, encompassing diverse conditions Therefore, this association should be interpreted as preliminary and hypothesis-generating rather than indicative of a specific biological mechanism. Abdominal circumference also showed a modest positive association with leukocyte counts. While these findings are physiologically plausible, the functional quality of leukocytes in individuals with metabolic disorders or obesity remains uncertain and may influence regenerative outcomes. Future studies with larger sample sizes and more homogeneous patient stratification are needed to determine whether specific comorbidities or metabolic parameters consistently influence the cellular composition of orthobiologics.

Several limitations pertaining to the study population should be acknowledged. Although all participants satisfied internationally recognized blood donation criteria, our cohort was characterized by a high prevalence of regular medication use (70.6%) and physician-diagnosed comorbidities (35.3%). This apparent discrepancy reflects a pragmatic, real-world sampling strategy: the average age of our participants (40.9 years) corresponds to a demographic in which antihypertensive agents, antidepressants, and hormonal therapies are commonly prescribed. It is important to clarify the nature of the statistical control applied in this study. The presence of comorbidities was included as a binary covariate in all LMMs, thereby adjusting outcome estimates for the average effect of having at least one diagnosed condition. This constitutes a statistical control, not an experimental one meaning that we can estimate the association between comorbidity status and product composition, but we cannot isolate causal mechanisms or disentangle the effects of specific diseases or their pharmacological treatments. We lacked the statistical power and detailed pharmacological phenotyping necessary to examine the effects of specific drug classes or disease severities on platelet count, leukocyte composition, and growth factor content. The medication variable, given the extensive heterogeneity of drug classes (antihypertensives, antidepressants, hormonal therapies) and the limited sample size, could not be meaningfully incorporated as a covariate without risking model overfitting and loss of interpretability. Certain agents represented in our sample—notably selective serotonin reuptake inhibitors and angiotensin receptor blockers—have been reported to influence platelet activation and secretory responses, and their potential to confound the biological quality of orthobiologics remains an open and important question. Therefore, these findings should be interpreted as deriving from a pragmatic, real-world cohort rather than a strictly controlled healthy reference population, and the comorbidity adjustment should be understood as a partial, exploratory mitigation rather than a complete solution to this source of variability. Future studies with larger, more homogeneous cohorts and detailed medication histories are warranted to disentangle these potential confounders from protocol-dependent effects. An inherent and important limitation of this comparative analysis is the discrepancy in initial blood volumes collected for PRP (28 mL) versus i-PRF (36 mL), which reflects the distinct clinical protocols and tube systems employed in routine practice. This difference directly influences the absolute final volume produced and, consequently, total cellular delivery. To mitigate this confounding, we have emphasized two normalized metrics: (i) percentage volume recovery, which showed that i-PRF yields a higher proportion of the initial blood volume as injectable product (10.16% vs. 22.3%), and (ii) absolute total platelet and leukocyte yields, which represent the complete cellular payload obtained from each preparation. When interpreted through these normalized measures, the comparison shifts from one of simple concentration to one of overall biological delivery capacity. Nonetheless, readers should view the contrast between PRP and i-PRF as a protocol-level performance comparison governed by the specific centrifugation parameters, tube selection, and starting volumes of each technique rather than as an intrinsic biological property of the platelet concentrate per se. Future head-to-head studies using identical starting volumes would be valuable to isolate the biological effects of the preparation method independent of volumetric factors. These findings indicate that PRP and i-PRF exhibit distinct biological profiles in terms of cellularity, volume, and total PDGF-BB content. Whether these laboratory differences translate into distinct clinical outcomes remains unknown. Well-designed randomized controlled trials are essential to clarify potential the clinical implications of these differences and to guide evidence-based, patient-tailored application of PRP and i-PRF in musculoskeletal disorders such as tendinopathies and osteoarthritis.

This study has several limitations that should be acknowledged. It is important to note that our biological characterization is limited to PDGF-BB and cellular composition; no other cytokines or functional assays were performed. First, its laboratory and cross-sectional design precludes any direct inference regarding clinical efficacy, biological activity in vivo, or therapeutic superiority between PRP and i-PRF.

In addition, i-PRF is typically produced from smaller volumes (to prevent premature clotting), direct comparisons of absolute delivery (total protein/cell count) are skewed by the preparation technique rather than the biological potential of the patient’s blood. Although cellular composition, volume, and total PDGF-BB content provide important surrogate markers, they do not capture the complex interactions occurring after injection, including tissue-specific responses, fibrin remodeling, cytokine release kinetics, and immune modulation. Second, PDGF-BB was assessed at a single timepoint, which likely underestimates the biological potential of i-PRF, given its fibrin-based structure and sustained growth factor release profile; therefore, cumulative or time-dependent release was not evaluated. Third, PDGF-BB was quantified in non-activated samples. Therefore, the values reflect total content rather than the bioactive released following physiological activation. The higher levels in PRP indicate a greater reservoir, but clinical efficacy also depends on the temporal release profile. A broader multiplex cytokine panel was initially planned; however, the fibrin clot formation inherent to i-PRF caused persistent technical interference with flow cytometry-based assays, precluding reliable quantification. We therefore restricted the analysis to PDGF-BB as a robust, consistently measurable marker of platelet degranulation. Consequently, the biological characterization remains confined to a single growth factor, and the broader secretome of these products was not captured, representing an important direction for future studies. Additionally, the sample consisted predominantly of female and Caucasian participants, which may limit generalizability to other populations. Although we statistically controlled for sex and age in our analyses, physiological hematological differences between men and women may influence not only product volume, as observed, but also functional aspects not measured in this study, such as growth factor release kinetics or the profile of inflammatory cytokines. Fifth, our exploratory analyses suggesting associations between comorbidities and product characteristics (volume and leukocyte content) must be interpreted with caution. The comorbidity group was relatively small and clinically heterogeneous, including conditions with potentially distinct effects on hematological parameters. While statistically significant in our models, these associations may reflect confounding factors or type I error due to multiple testing, and they do not establish causality. Finally, although the protocols were carefully standardized, the findings are specific to the preparation techniques employed and should not be extrapolated to other PRP or i-PRF systems. These limitations reinforce the need for future studies integrating longitudinal growth factor analyses, functional assays, and well-designed randomized clinical trials to clarify the clinical implications of the observed biological differences.

## 5. Conclusions

Within the parameters assessed, such as cellular composition, volume, and immediate PDGF-BB availability, PRP and i-PRF demonstrate distinct profiles. However, given the multifactorial nature of platelet-derived bioactivity, these differences should be interpreted in the context of a limited growth factor analysis. A comprehensive understanding of their relative biological potential will require further studies incorporating a broader panel of mediators and release kinetics.

PRP is characterized by higher platelet concentration and greater immediate availability of PDGF-BB, whereas i-PRF provides larger volumes and a higher absolute delivery of leukocytes, reflecting differences in processing and fibrin structure. These findings indicate that PRP and i-PRF may not be interchangeable therapies. These laboratory findings underscore the need for product-specific protocols in future clinical research. Direct translation of these in vitro profiles to clinical superiority requires validation in randomized controlled trials.

## Figures and Tables

**Table 1:** Categorical and descriptive variables of study participants (n = 34).

Variable	n (%) or Mean ± SD
Sex (female)	26 (76.5)
Race	
- Caucasian	33 (97.1)
- Black	1 (2.9)
BMI category	
- Normal Weight	14 (41.2)
- Overweight	14 (41.2)
- Obesity Grade I	3 (8.8)
- Obesity Grade II	2 (5.9)
- Morbid Obesity	1 (2.9)
Smoking (yes)	1 (2.9)
Alcohol consumption (yes)	17 (50.0)
Regular medication use (yes)	24 (70.6)
Presence of comorbidities (yes)	12 (35.3)
Age (years)	40.9 ± 9.8
Body weight (kg)	76.1 ± 16.6
Abdominal circumference (cm)	86.4 ± 14.1
Body mass index (kg/m^2^)	26.8 ± 4.94

Data are presented as number (percentage) or mean ± standard deviation (SD). BMI: body mass index.

**Table 2:** Description of baseline samples and biological products in terms of cellularity, total PDGF-BB content, and volume. Normalization of PDGF-BB according to platelet number.

Variable	Baseline	PRP	i-PRF	Comparison Note
Platelets (cells/mm^3^)	231,706 ± 46,270	1,416,824 ± 307,679	421,853 ± 101,805	PRP is ~3.3× more concentrated
Leukocytes (cells/mm^3^)	6432 ± 1398	19,649 ± 6524	11,904 ± 2349	—
PDGF-BB (pg/mL)	—	126,186 ± 49,414	9471 ± 5639	Raw value shows 13× difference
PDGF-BB per 10^6^ Platelets	—	89.06 pg	22.45 pg	The Normalized Benchmark
Volume (mL)	—	2.84 ± 0.48	7.60 ± 1.97	—
Total platelets (cells)	—	4.03 × 10^9^ ± 1.10 × 10^9^	3.32 × 10^9^ ± 1.37 × 10^9^	Statistical parity in total payload
Total leukocytes (cells)	—	5.51 × 10^7^ ± 2.04 × 10^7^	9.07 × 10^7^ ± 3.35 × 10^7^	—
Volume recovery (%) *		10.16 ± 1.73	22.34 ± 5.79	

Notes: Data are presented as mean ± standard deviation. PRP: platelet-rich plasma; i-PRF: injectable platelet-rich fibrin; PDGF-BB: platelet-derived growth factor–BB. * Volume Recovery (%): (Final volume per participant, in mL/Fixed initial blood volume, in mL) × 100.

**Table 3:** LMM results and 95% confidence intervals (CIs) for associations between platelet count and independent variables. Independent variables included group, sex, comorbidity, alcohol consumption, abdominal circumference, and age.

Variables	Estimate ^b^ (SE)	CI 95%	df	T	*p*	Tolerance (VIF)
(Intercept)	676,691 (31,191)	614,742–738,639	28	21.695	<0.001 *	
Group: PRP-Basal ^a^	1.19 × 10^6^ (38,791)	1.11 × 10^6^–1.26 × 10^6^	66	30.551	<0.001 *	1.00 (1.00)
Group: i-PRF-Basal ^a^	190,147 (38,791)	113,105–267,190	66	4.902	<0.001 *	
Age	3117 (3041)	−2922–9156	28	1.025	0.314	0.84 (1.19)
Sex: M-F ^a^	−23,252 (68,229)	−158,761–112,256	28	−0.341	0.736	0.85 (1.17)
Comorbidity: Yes-No ^a^	−49,516 (54,498)	−157,754–58,721	28	−0.909	0.371	0.94 (1.05)
Abdominal circumference	−846 (2051)	−4919–3227	28	−0.413	0.683	0.86 (1.16)
Alcohol Consumption: Yes–No ^a^	−32,468 (50,800)	−133,361–68,425	28	−0.639	0.528	0.97 (1.03)

Notes: ^a^ Reference category indicated after variable name; ^b^ beta coefficient; * statistically significant at *p* < 0.05.

**Table 4:** LMM results and 95% confidence intervals (CIs) for associations between leukocyte count and independent variables.

Variables	Estimate ^b^ (SE)	CI 95%	df	t	*p*	Tolerance (VIF)
(Intercept)	12,159.5 (777)	10,608.1–13,711	66	15.65	<0.001 *	-
Group: PRP-Basal ^a^	13,216.5 (1022.1)	11,175.8–15,257	66	12.93	<0.001 *	1.00 (1.00)
Group: i-PRF-Basal ^a^	5472.4 (226.6)	5020–5925	66	24.15	<0.001 *	
Comorbidity: Yes–No ^a^	1302 (1129.7)	−1012–3616	28	1.15	0.259	0.94 (1.05)
Sex: M-F ^a^	−2620.6 (1414.3)	−5517.6–276	28	−1.85	0.074	0.85 (1.17)
Alcohol Consumption: Yes–No ^a^	1609.8 (1053)	−547.2–3767	28	1.53	0.138	0.97 (1.03)
Abdominal Circumference	73.6 (42.5)	−13.4–161	28	1.73	0.094	0.86 (1.16)
Age	79.5 (63)	−49.6–209	28	1.26	0.218	0.84 (1.19)

Notes: ^a^ Reference category indicated after variable name; ^b^ beta coefficient; * statistically significant at *p* < 0.05.

**Table 5:** LMM results and 95% confidence intervals (CIs) for the associations between PDGF-BB quantification and the independent variables Group, Sex, Comorbidity, Alcohol consumption, Age and Abdominal circumference.

Variables	Estimate ^b^ (SE)	CI 95%	df	t	*p*	Tolerance (VIF)
(Intercept)	67,696 (5744)	56,203–79,189	28	11.78	<0.001 *	-
Group: i-PRF-PRP ^a^	−116,715 (8266)	−133,249–−100,181	33	−14.12	<0.001 *	1.00 (1.00)
Abdominal circumference	106 (378)	−650–862	28	0.28	0.78	0.74 (1.43)
Comorbidity: Yes–No ^a^	11,054 (10,036)	−9027–31,135	28	1.10	0.28	0.89 (1.11)
Sex: M-F ^a^	−6640 (12,564)	−31,780–18,501	28	−0.52	0.60	0.72 (1.37)
Alcohol Consumption: Yes–No ^a^	−4265 (9355)	−22,983–14,454	28	−0.34	0.733	0.94 (1.06)
Age	427 (560)	−693–1548	28	0.76	0.45	0.70 (1.42)

Notes: ^a^ Reference group; ^b^ beta coefficient; * statistical significance.

**Table 6:** LMM results and 95% confidence intervals (CIs) for the associations between volume quantification (mL) and the independent variables Group, Sex, Comorbidity, Alcohol consumption, Age and Abdominal circumference.

Variables	Estimate ^b^ (SE)	CI 95%	df	T	*p*	Tolerance (VIF)
(Intercept)	5.00 (0.21)	4.57–5.44	28	23.336	<0.001 *	-
Group: i-PRF-PRP ^a^	4.75 (0.28)	4.19–5.31	33	16.913	<0.001 *	1.00 (1.00)
Alcohol Consumption: Yes–No ^a^	0.21 (0.35)	−0.48–0.92	28	0.456	0.54	0.94 (1.06)
Comorbidity: Yes–No ^a^	0.93 (0.37)	0.17–1.68	28	2.248	0.02 *	0.89 (1.11)
Sex: M-F ^a^	−1.32 (0.47)	−2.27–−0.37	28	−3.233	0.009 *	0.72 (1.37)
Abdominal Circumference	0.04 (0.01)	0.01–0.07	28	2.784	0.006 *	0.74 (1.34)
Age	−0.02 (0.02)	−0.06–0.01	28		0.28	0.70 (1.42)

Notes: ^a^ Reference; ^b^: beta coefficient; * statistical significance.

**Table 7:** LMM results and 95% confidence intervals (CIs) for the associations between total platelet counts and the independent variables Group, Comorbidity, Age, Sex, Alcohol consumption, and Abdominal circumference.

Variables	Estimate ^b^ (SE)	CI 95%	df	t	*p*	Tolerance (VIF)
(Intercept)	3.49 × 10^9^ (2.55 × 10^8^)	2.98 × 10^9^–4.00 × 10^9^	28	13.69	<0.001 *	
Group: i-PRF-PRP ^a^	−7.06 × 10^8^ (1.45 × 10^8^)	−9.96 × 10^8^–−4.15 × 10^8^	1.82 × 10^35^	−4.86	<0.001 *	1.00 (1.00)
Alcohol Consumption: Yes–No ^a^	2.86 × 10^7^ (4.15 × 10^8^)	−8.01 × 10^8^–8.59 × 10^8^	28	0.06	0.94	0.94 (1.06)
Comorbidity: Yes–No ^a^	4.19 × 10^8^ (4.45 × 10^8^)	−4.71 × 10^8^–1.31 × 10^9^	28	0.94	0.35	0.89 (1.11)
Abdominal circumference	2.78 × 10^7^ (1.67 × 10^7^)	−5.69 × 10^6^–6.13 × 10^7^	28	1.66	0.10	0.74 (1.34)
Sex: M-F ^a^	−9.37 × 10^8^ (5.57 × 10^8^)	−2.05 × 10^9^–1.80 × 10^8^	28	−1.67	0.10	0.72 (1.37)
Age	−8.95 × 10^6^ (2.48 × 10^7^)	−5.86 × 10^7^–4.07 × 10^7^	28	−0.36	0.72	0.70 (1.42)

Notes: ^a^ Reference category indicated after variable name; ^b^ beta coefficient; * statistically significant at *p* < 0.05.

**Table 8:** LMM results and 95% confidence intervals (CIs) for associations between total leukocyte count and the independent variables Group, Comorbidity, Age, Sex, Alcohol consumption, and Abdominal Circumference.

Variables	Estimate ^b^ (SE)	CI 95%	df	t	*p*	Tolerance (VIF)
(Intercept)	7.06 × 10^7^ (4.72 × 10^6^)	6.11 × 10^7^–8.00 × 10^7^	28	14.94	<0.001 *	
Group: i-PRF–PRP ^a^	3.55 × 10^7^ (3.69 × 10^6^)	2.82 × 10^7^–4.29 × 10^7^	2.05 × 10^29^	9.62	<0.001 *	1.00 (1.00)
Alcohol Consumption: Yes–No ^a^	8.99 × 10^6^ (7.69 × 10^6^)	−6.40 × 10^6^–2.44 × 10^7^	28	1.16	0.25	0.94 (1.06)
Abdominal Circumference	1.01 × 10^6^ (310,498)	389,746–1.63 × 10^6^	28	3.25	0.003 *	0.74 (1.34)
Comorbidity: Yes–No ^a^	2.14 × 10^7^ (8.25 × 10^6^)	4.94 × 10^6^–3.80 × 10^7^	28	2.59	0.01 *	0.89 (1.11)
Age	−208,247 (460,373)	−1.13 × 10^7^–712,957	28	−0.45	0.65	0.70 (1.42)
Sex: M–F ^a^	−2.09 × 10^7^ (1.03 × 10^7^)	1.03 × 10^7^–−4.15 × 10^7^	28	−2.01	0.053	0.72 (1.37)

Notes: ^a^ Reference category; ^b^ B: beta coefficient; * statistical significance.

## Data Availability

The data presented in this study are available on request from the corresponding author. The data are not publicly available due to ethical and privacy restrictions.

## Abbreviations

The following abbreviations are used in this manuscript:

ACD	Acid citrate dextrose
AIC	Akaike Information Criterion
ANVISA	Brazilian Health Regulatory Agency (Agência Nacional de Vigilância Sanitária)
BMI	Body mass index
CAAE	Certificate of Presentation for Ethical Consideration (Certificado de Apresentação para Apreciação Ética)
CBC	Complete blood count
CI	Confidence interval
ELISA	Enzyme-linked immunosorbent assay
LMM	linear mixed model
HCPA	Hospital de Clínicas de Porto Alegre
i-PRF	Injectable platelet-rich fibrin
IRB	Institutional Review Board
L-PRP	Leukocyte-rich platelet-rich plasma
PDGF	Platelet-derived growth factor
PDGF-BB	Platelet-derived growth factor BB isoform
PRF	Platelet-rich fibrin
PRP	Platelet-rich plasma
RDC	Collegiate Board Resolution (Resolução da Diretoria Colegiada)
SD	Standard deviation
VIF	Variance inflation factor
WHO	World Health Organization
